# Atopic Dermatitis: Epidemiology and Clinical Phenotypes

**DOI:** 10.5826/dpc.1104a146

**Published:** 2021-10-01

**Authors:** Annunziata Raimondo, Serena Lembo

**Affiliations:** 1 Department of Medicine, Surgery and Dentistry, “Scuola Medica Salernitana”, University of Salerno, Salerno, Italy

**Keywords:** atopic dermatitis, epidemiology, phenotypes, clinical features

## Abstract

Atopic dermatitis (AD) is a chronic, lifelong, relapsing condition. The wide spectrum of the possible clinical presentations, depending on patient’ s age, age of onset of disease, topography and morphology of dermatitis, limits the epidemiologic information on its prevalence and incidence. A clear definition of the different clinical AD phenotypes and epidemiology is essential for an appropriate patient’s treatment and management, in particular for adults. This review summarizes the most recent epidemiologic data from the 21^st^ century, on AD prevalence and incidence rates either in children or adults, with a special focus on their trends in Europe. Moreover, an effort to categorize diverse AD clinical expressions, has been made, aiming to facilitate differential diagnosis and speed up the start of the correct therapy.

## Epidemiology of Atopic Dermatitis: Data From the 21^st^ Century

Updated prevalence and incidence data of AD, across different age groups and countries, increase our understanding of the disease burden. It is well established that in most cases (approximately 80%) AD onset occurs during the first years of life, with frequent remissions in adolescence (approximately 60% of individuals). Recently, some studies have reported an adult-onset AD, even if epidemiological and clinical features of this adult form need to be further clarified [1–4]. AD incidence and prevalence register a stable plateau in Europe and North America, while they are increased in other continents, such as Asia. There are few recent studies on the incidence of AD. Most of them have been conducted in Europe (EU) and USA [5]. Limited information on the prevalence and incidence of AD among adults suggest the wide variability that may be dependent by the population, disease definitions, diagnostic criteria, presence or not of disease register, and lack of a universally accepted index for disease severity. Future studies with more standardized methods need to be conducted to assess epidemiology of AD, especially for adults: they are important to improve healthcare planning and patient management.

### AD in Children: Prevalence and Incidence

The point prevalence (the proportion of the population that has the disease at a specific point in time) ranged from 0% (Nigeria) to 18.2% (Turkey) [6,7]. The 1-year period prevalence (the proportion of the population presenting the disease for 1 year) ranged from 4.1% to 22.7%. The 1-year prevalence of doctor diagnosed (the proportion of the population with the disease diagnosed by a doctor in 1 year) ranged from 0.96% to 22.6%, and the lifetime symptom prevalence (the proportion of population that has the disease symptoms at least once in a lifetime) ranged from 4.4% to 17.7% assessed at age 7–15 years [8–12]. The 1-year incidence (the annual incidence, the probability of the disease occurrence in the population) in children ranged from 10.2 per 1,000 person years in Italy (95% Confidence Interval (CI), 9.9–10.6) to 95.6 per 1,000 person years in Scotland (CI 93.4–97.9 %). Many studies reported that the highest incidence of AD occurred during infancy, with a disease onset by the age of 7 years [1,13,14]. The incidence was also high in early childhood during the first 18 months of life [13,15].

### AD in Adults: Prevalence and Incidence

In the overall population, the 1-year adult prevalence of AD was 4.9% (95% CI: 4.6% – 5.2%) in the US, 3.5% (95% CI: 3.1%–3.9%) in Canada, and 4.4% (95% CI: 4.2%–4.6%) in Europe (EU) [16,17]. The 1-year prevalence of diagnosed AD ranged from 1.2% (Asia) to 17.1% (EU) [8,9,18]. The lifetime symptom prevalence ranged from 3.0% to 17.7% [8,9,12]. The point prevalence of adult AD was reported to be 2.9% in Japan, with 1-year rate of 3.0% and lifetime prevalence of 3.3% [19]. A significant incidence was also reported during adolescence and adulthood. Studies recorded an incidence rate of AD in adults of 7.41 per 1,000 person years (6.27–8.74) [20], and a proportion of adult onset of 8.0% in Germany at age 28–30 years [16,21,22–25].

### Trends of Prevalence by Sex

Both the 1-year prevalence and lifetime prevalence of diagnosed AD were higher in females (range 0.6–24.3%; 1.0–35.5%, respectively) than in males (range 0.8–17.6%; 1.4–37.3%, respectively), except for the UK, where the prevalence was the same (2.5%), and the US, where prevalence was numerically, but not significantly, higher in males (5.1% vs 4.6%) [5,26].

### Spotlight on AD Epidemiology in Europe

European trends seem to be in-line with those reported from global studies: AD is more prevalent in children compared to adults, and in overcrowded urban areas [27]. The prevalence in adolescent group is between 1.5% (Lithuania) and 15% (Bulgaria, Denmark, Finland, and Hungary). Epidemiology in adult group remains a challenge. An international, cross sectional, web-based survey was performed in 2018 [26]. It reported 1-year adult prevalence of AD in EU of 4.4% (95% CI: 4.2%–4.6%) with country ranges from 2.2% (95% CI: 1.9%–2.5%) in Germany to 8.1% (95% CI: 7.5%–8.6%) in Italy. Italy and Spain reported a higher point adult prevalence respect to other countries. The prevalence in females was significantly enhanced in Spain (9.3% females vs 5.1% males, P < .05). France, Italy, and Spain had more mild forms of adult AD compared with the ones reported in UK and Germany. Italy had an important regional variability, showing higher adult prevalence rates in Mediterranean regions [28]. The reasons of this variability are many: genetic, behavioral or cultural components, socioeconomic conditions, and climatic factors [29]. In general, mild, or moderate severity were the most common clinical presentations, with low proportions of severe form.

## The Thousand Faces of AD: The Wide Spectrum of Clinical Phenotypes

The heterogeneous and intriguing clinical aspects of AD reflects the complex nature of this lifelong disease. Traditionally, clinical lesions are classified as *“acute”*, characterized by oozing, edema, and erythema, or *“chronic”*, with prevalent xerosis, lichenification, and dyspigmentation. However, as chronic relapsing condition, both types of lesions can coexist in the same individual, especially during flares. The main hallmark of AD is pruritus, responsible for excoriations and skin lichenification. A clear definition of the different clinical AD phenotypes (Table 1) is essential to improve its treatment and management, passing from a “one-size-fits-all” to a personalized approach based on differentiation of AD clinical expressions.

### Age-Related Clinical Phenotype

Many clinical pictures of AD have been described based on the age of the patient: infantile AD (3 months/2 years), childhood AD (2–12 years), adolescent/adult AD (12–60 years), and elderly AD (> 60 years). Pruritus remains the hallmark in all stages, except for very initial disease onset (< 3 months). In the infantile form, eczematous lesions typically affect scalp, cheeks, neck, and extensor parts of the extremities with edematous papulo-vesicles, oozing, and crusting. In the childhood stage both acute and chronic lesions are present. Popliteal, antecubital fossa and hand are predilected areas involved. In adolescents and adults, eczematous lesions prevalently affect flexural areas, neck, and head. Periorbital areas are also involved, mainly in females. Sometimes, an erythrodermic status could occur. Elderly AD is an underestimated clinical phenotype characterized by extensive eczematous lesions, also presenting with erythrodermic aspect. Three forms of elderly-type AD have been recognized: elderly onset, relapsing, and continuous subtype. Elderly AD phenotype poses many problems of differential diagnosis that might mimic AD, such as allergic contact dermatitis or cutaneous-T cell lymphoma, and it needs to be deeply investigated to avoid misdiagnosis.

### Topography-Related Clinical Phenotypes

#### Head and Neck Dermatitis

Recently, a specific topographical form of AD has been investigated: it is the Head and Neck dermatitis (HND), also called “portrait dermatitis”, characterized by erythematous and scaly plaques localized on the face and neck [30,31]. There are many other possible causes for HND, including allergic contact dermatitis to topical products, topical corticosteroid withdrawal syndrome, aeroallergen sensitization, rosacea, seborrheic dermatitis, and sensitization to Malassezia furfur. Interestingly, all these potential causes may coexist with AD in the same patient. Reports show that elevated serum levels of Malassezia-specific IgE in HND, and positive response to systemic antifungals, may support the clinical diagnosis.

#### Scalp

It is frequently involved with xerotic, scaly, erythematous, and sometimes lichenified plaques.

#### Facial Dermatitis

It is very common in AD in all stages, from infants to adults. In many cases this is the only clinical presentation of the disease.

#### Eyelids

Eyelids of AD patients are characterized by lichenification, depigmentation, and loss of lashes, accompanied by itch and burn. Moreover, AD is associated with ocular diseases, including conjunctivitis and cataracts.

#### AD lips

When lips are affected by AD, these appear red and dry. Sometimes, a median fissure of the lower lip and angular cheilitis with alongside lateral fissures are visible.

#### Flexural involvement

This is characterized by erythema, edema, excoriation, lichenification, oozing, and crusting. Usually, affected flexural areas are neck, cubital and popliteal fossae, wrists, and ankles. It is more prevalent in adolescent and adult Caucasian patients with a chronic persisting course.

#### Nipple Dermatitis

Nipples and areolas involvement is found in 11–23% of AD patients. It is frequent in post-puberal girls and young adults, and it can be triggered or aggravated by breast-feeding. Nipple dermatitis is typically symmetrical. Its specificity as minor diagnostic feature of AD remains to be clarified.

#### Hand and Foot AD

This form appears with xerotic, scaly, lichenified, and fissured skin, notably on the dorsal part. This phenotype is more common in adulthood, especially in females. Conditions, such as juvenile palmoplantar dermatitis or dermatitis plantaris sicca, have been described in children, with a possible link to atopic diathesis. The risk of hand dermatitis was greater in children with persistent or severe AD. Dyshidrotic eczema may be a clinical phenotype of hand and foot AD that manifests as vesicles and blisters on the palms and soles.

### Morphology-Related Clinical Phenotypes

#### Nummular phenotype

The term derives from the coin like appearance of the lesions. Indeed, they are typically circinate and ovoid plaques with central clearing and peripheral extension of papules and papulo-vesicles. Lower extremities are predominantly affected. It is the most common morphologic variant of AD, and it is more prevalent in children and adult-onset forms. However, if nummular eczema is AD in all cases needs to be deeply clarified.

#### Prurigo Nodularis phenotype

In some AD patients the morphology of lesions is characterized by multiple excoriated hyperkeratotic and intensely itchy nodules. A condition defined Prurigo Nodularis secondary to AD. It is more common in adults. Also, for this morphologic variant an accurate differential diagnosis must be considered.

#### Erythrodermic phenotype

It is the presence of erythema on > 90% of the body surface area. This form is frequent in adolescents and adults, especially in those with a life-long disease.

#### Lichenified phenotype

In this variant the skin is thick with accentuated creases and a leathery appearance. It is more common in adolescents and adults from South-East Asia or Africa than in Caucasian patients.

#### Follicular/papular phenotype

It is a morphological subtype more frequent in dark skin, characterized by papular-lichenoid lesions.

## Conclusions and Open Questions

AD is a heterogeneous disease that can be classified according to many and different criteria, based on morphology, topography, severity, age at onset, or disease course.

The difficulty in identifying AD, for the lack of validated universal diagnostic criteria as well as for the variegated clinical phenotypes, is responsible for the approximation of the epidemiology of this disease. In fact, AD clinical phenotypes and epidemiology clarification are current challenges for dermatologists. It will be useful to realize a practical guide to distinguish the main features of any clinical phenotypes to perform an earlier diagnosis with more appropriate treatment and management decisions. The variability of AD implicates the need for a personalized therapy. Establishing an association between phenotype and treatment response, sheds light on the different pathogenetic mechanisms that express in distinct clinical presentations and require diverse therapeutic strategies.

## Figures and Tables

**Table 1:** Clinical Phenotypes of AD and Related-Differential Diagnosis.

PHENOTYPE	CLINICAL FEATURES	DIFFERENTIAL DIAGNOSIS

**Age-related clinical phenotype**		

Infantile (0–2 years)	Eczematous lesions typically affect scalp, checks, neck, and extensor parts of the extremities with edematous papulo-vesicles, oozing, and crusting.	Seborrheic dermatitis, psoriasis, scabies, ichthyosis vulgaris, phenylketonuria Genetic syndromes: Di George syndrome, Netherton syndrome, Wiskott-Aldrich syndrome.

Childhood (2–12 years)	Eczematous lesions typically affect popliteal and antecubital fossa, hand, and foot, with edematous papulo-vesicles, oozing, crusting, and lichenification.	Impetigo, psoriasis, tinea manuum, pedis.

Adolescent/adult (12–60 years)	Eczematous lesions prevalently affect head, neck and flexural areas, with xerosis, lichenification, and depigmentation. In females they also involve periorbital and nipple areas.	ACD, psoriasis, cutaneous T-cell lymphoma, pityriasis rubra pilaris, pityriasis rosea, asteatotic eczema.

Elderly (>60 years)	Extensive eczematous lesions, including flexural areas, up to erythrodermic aspect.	ACD, psoriasis, cutaneous T-cell lymphoma, pityriasis rubra pilaris, pityriasis rosea, asteatotic eczema.

**Topography-related clinical phenotypes**		

Head and neck		

Scalp	Erythema, scaling, crusting, lichenification, excoriation, and scarring.	Psoriasis, seborrheic dermatitis, ACD, ICD, tinea capitits.
	
Face	Erythema, oozing, edema, xerosis, lichenification, dyspigmentation, and excoriation.	ACD, ICD, psoriasis, seborrheic dermatitis, impetigo.
	
Eyes	Erythema, scaling, crusting, lichenification, depigmentation, and scarring.	ACD, ICD, psoriasis, infectious conjunctivitis.
	
Lips	Erythema, xerosis, lichenification, fissuration, and dyspigmentation.	ACD, ICD, psoriasis, infectious cheilitis.
	
Flexures	Erythema, edema, excoriation, lichenification, oozing, and crusting.	Psoriasis, seborrheic dermatitis, ACD, ICD, infectious intertrigo, scabies.

Nipples	Erythema, scaling, crusting, lichenification, excoriation, and scarring.	Psoriasis, ACD, Jogger’s nipple, Paget’s disease.

Hand and foot	Erythema, xerosis, lichenification, scaling, crusting, fissuration, and dyspigmentation.	Psoriasis, ACD, ICD, tinea manuum, tinea pedis.

**Morphology-related clinical phenotypes**		

Nummular	Circinate and ovoid plaques with central clearing and peripheral extension of papules and papulo-vesicles.	ACD, tinea corporis, psoriasis, pityriasis rosea, asteatotic eczema.

Prurigo Nodularis	Excoriated hyperkeratotic and intensely itchy nodules.	Scabies, cutaneous T-cell lymphoma, psoriasis bullous pemphigoid, paraneoplastic manifestations.

Erythrodermic phenotype	Erythema on >90% of the body surface area.	Cutaneous T-cell lymphoma, psoriasis, bullous pemphigoid, Lyell syndrome, paraneoplastic manifestations.

Lichenified	Skin is thick with accentuated creases and a leathery appearance.	Psoriasis, cutaneous T-cell lymphoma, ACD.

Follicular/papular	Papular-lichenoid lesions.	Lichen ruber planus, psoriasis, pityriasis rubra pilaris.

ACD = allergic contact dermatitis; ICD = irritant contact dermatitis.
